# How risky is a second allogeneic stem cell transplantation?

**DOI:** 10.1038/s41375-024-02318-3

**Published:** 2024-06-25

**Authors:** Olaf Penack, Mouad Abouqateb, Christophe Peczynski, William Boreland, Nicolaus Kröger, Robert Zeiser, Fabio Ciceri, Thomas Schroeder, Peter Dreger, Jakob Passweg, Johannes Schetelig, Matthias Stelljes, Igor Wolfgang Blau, Georg-Nikolaus Franke, Katarina Riesner, Hélène Schoemans, Ivan Moiseev, Zinaida Peric

**Affiliations:** 1 Medical Clinic, Department for Haematology, Oncology and Tumorimmunology, Charité Universitätsmedizin Berlin, Berlin, Germany; 2 EBMT Transplant Complications Working Party, Paris, France; 3 Department of Stem Cell Transplantation, University Medical Center Hamburg, Hamburg, Germany; 4 Department of Medicine I, Medical Center-University of Freiburg, Faculty of Medicine, Freiburg, Germany; 5 IRCCS Ospedale San Raffaele s.r.l, Milano, Italy; 6 University Hospital Essen, Essen, Germany; 7 University Hospital Heidelberg, Heidelberg, Germany; 8 University Hospital Basel, Basel, Switzerland; 9 University Hospital TU Dresden, Dresden, Germany; 10 University of Muenster, Muenster, Germany; 11 Medical Clinic and Policinic 1, Leipzig, Germany; 12 Department of Hematology, University Hospitals Leuven and KU Leuven, Leuven, Belgium; 13 RM Gorbacheva Research Institute, Pavlov University, St Petersburg, Russia; 14 Department of Haematology, University Hospital Centre Rijeka, Rijeka, Croatia

**Keywords:** Translational research, Leukaemia

## Abstract

There is no consensus on second allogeneic stem cell transplantation (alloSCT) indications in patients with hematologic malignancies relapsing after a first alloSCT. In historic publications, a very high non-relapse mortality (NRM) has been described, arguing against performing a second alloSCT. We analysed the outcome of 3356 second alloSCTs performed 2011–21 following a hematologic malignancy relapse. Outcomes at two years after second alloSCT were: NRM 22%, relapse incidence 50%, overall survival 38%, and progression-free survival 28%. Key risk factors for increased NRM were: older age, low performance score, high disease-risk-index, early relapse after the first alloSCT, unrelated/haploidentical donor, and GVHD before second alloSCT. Any type of GVHD after first alloSCT was also important risk factor for acute GVHD and chronic GVHD after second alloSCT. There was a preferential use of a different donor (80%) at second alloSCT from first alloSCT. However, in multivariate analysis, the use of the same alloSCT donor for second alloSCT vs. a different donor was not associated with any of the survival or GVHD endpoints. We show considerably improved outcome as compared to historic reports. These current data support a wider use of second alloSCT and provide risk factors for NRM that need to be considered.

## Introduction

Relapse of the underlying malignancy after the first allogeneic stem cell transplantation (alloSCT) is a frequent problem, usually leading to consideration of a subsequent alloSCT, especially in younger and healthier patients. A previous European Society for Blood and Marrow transplantation (EBMT) analysis including patients with second transplants before 2009 found a high treatment-related mortality incidence of ~40% after second alloSCT [1]. Subsequently, similar outcome data was published in disease-specific subgroups [2–5]. These historic data showing high treatment-related mortality is the current basis for decision-making and argue against a second alloSCT in many patients.

We asked the question if this published data is still valid and up to date enough to be the foundation for clinical decision-making. Based on recent advances in the field, our assumption was that non-relapse mortality (NRM) after second alloSCT is considerably lower nowadays. Since there are no randomized studies or comprehensive recent large data file analyses available, we performed a retrospective EBMT data set analysis. We collected relevant data on second alloSCT-related mortality and other major outcome parameters, such as incidence of graft-versus-host disease (GVHD), relapse rates, and causes of deaths. Furthermore, we describe procedures of conditioning, as well as GVHD prophylaxis, which often differ between the first and second alloSCT. Our current analysis is intended to provide the data, which is required for evidence-based decision-making of a second alloSCT in patients with hematologic malignancies.

## Patients and methods

### Study design and data collection

This is a retrospective multicenter analysis using the data set of the EBMT registry. The EBMT is a voluntary working group of more than 600 transplant centers which are required to report regular follow-up on all consecutive stem cell transplantations. Audits are routinely performed to determine the accuracy of the data. The study was planned and approved by the Transplant Complications Working Party of the EBMT. All patients gave their written informed consent to use their personal information for research purposes. The study was conducted in accordance with the Declaration of Helsinki and Good Clinical Practice guidelines.

### Eligibility criteria

Inclusion criteria:

- Patients who underwent a second alloSCT between 2011 and 2021.
- Types of donors included: identical sibling, matched unrelated donor, mismatched unrelated donor, and haploidentical donor.
- Stem cell sources: bone marrow and peripheral blood.
- Diagnoses: patients with hematologic malignancies including acute leukemia, chronic leukemia, myelodysplastic/myeloproliferative disorders, lymphoma, and plasma cell disorders.

Exclusion criteria:

- Patients with a previous autologous SCT (autoSCT).
- Non-calculable Disease Risk Index (DRI).
- Incoherent disease status between the first and second alloSCT.
- Evidence of non-engraftment of the first alloSCT.
- Patients without a relapse between the first and second alloSCT.
- Umbilical cord blood as donor source (due to low numbers).

### Data collection

Data collected included recipient and donor characteristics (age, sex, cytomegalovirus serostatus, and Karnofsky Performance Status (KPS) score), diagnosis, status at transplant, and transplant-related factors, including conditioning regimen, stem cell source, and GVHD prophylaxis. GVHD grading was performed according to published criteria for acute GVHD [6] and chronic GVHD [7]. For the purpose of this study, all necessary data were collected according to the EBMT guidelines, using the EBMT Minimum Essential Data forms.

### Statistical analysis

Median values and interquartile ranges, and minimum and maximum values were used to describe quantitative variables; frequency and percentage were used for categorical variables.

Study endpoints were NRM, overall survival (OS), progression-free survival (PFS), relapse incidence (RI), and incidence and severity of acute and chronic GVHD. The starting point of analyses was the date of second transplant for all endpoints. NRM was defined as death without relapse/progression, PFS was defined as survival without relapse or progression, RI was defined as disease recurrence. Probabilities of OS and PFS were calculated using the Kaplan–Meier method. Cumulative incidence was used to estimate NRM, RI, as well as acute and chronic GVHD in a competing risk setting, where death and relapse were considered as competing risks as appropriate [8]. Multivariate analyses were performed using the Cox cause-specific proportional-hazards model for all endpoints. All clinically known and potentially relevant risk factors were included in the multivariate models: patient age, year of transplant, patient and donor gender, donor to patient cytomegaly virus (CMV) combination, DRI, KPS, Donor Type, Same Donor in 1st alloSCT, stem cell source, GVHD between 1st and 2nd alloSCT, Delay between 1st alloSCT and 1st Relapse, Delay 1st Relapse to 2nd alloSCT, any level of total body irradiation (TBI) and conditioning intensity (reduced intensity conditioning (RIC) vs. myeloablative conditioning (MAC)). All factors were measured at the 2nd alloSCT. Center effect was taken into account by introducing a random effect or ‘frailty’ into all models. Results were expressed as the hazard ratio (HR) with the 95% confidence interval (95% CI). Statistical analyses were performed with R 4.3.0 software (R Development Core Team, Vienna, Austria) packages.

### Data sharing statement

Individual participant data will not be shared because patients agreed to data sharing with EBMT as well as with publication of results, but not to share data with third parties.

## Results

### Patient- and treatment characteristics

We identified 3356 second alloSCTs that met the inclusion criteria. Patient- and treatment characteristics of first- and second alloSCTs are shown in Table 1. The main underlying malignancies were AML 60%, ALL 15%, and MDS 8%.

**Table 1:** Baseline patient-, donor- and transplant-related characteristics at first- and second alloSCT.

*N* = 3356	First alloSCT	Second alloSCT
Age at transplant, yrs		
Median [Q1, Q3]	45.4 (31.9, 55.7)	48.2 (34.7, 58.7)
[Min, Max]	2.9–77.1	18.1–78.4
Age at diagnosis, yrs		
Median [Q1, Q3]	44.4 (30.9, 54.5)	
[Min, Max]	1.8–77.3	
Missing count	3	
Transplant year		
Median [Q1, Q3]	2014 (2011, 2017)	2016 (2014, 2019)
[Min, Max]	1986–2021	2011–2021
Patient sex at birth		
Male	1933 (57.6%)	
Female	1423 (42.4%)	
Cell source		
Peripheral blood	2778 (82.9%)	3083 (91.9%)
Bone marrow	507 (15.1%)	271 (8.1%)
Cord blood	60 (1.8%)	
Peripheral blood and cord blood	7 (0.2%)	
Bone marrow and cord blood	1 (0.03%)	
Missing count	3	2
Type of donor		
Identical sibling	1295 (38.6%)	593 (17.7%)
Matched unrelated 10/10	1025 (30.6%)	1188 (35.4%)
Mismatched unrelated 9/10	295 (8.8%)	309 (9.2%)
Unrelated (missing data on HLA)	701 (20.9%)	470 (14.0%)
Mismatched unrelated ≤8/10	34 (1.0%)	46 (1.4%)
Haploidentical HSCT	192 (5.7%)	750 (22.3%)
Missing count	3	0
Same donor as 1st alloSCT		
No		2321 (79.8%)
Yes		588 (20.2%)
Missing count		447
Karnofsky		
<90	624 (20.4%)	1258 (40.4%)
≥90	2442 (79.6%)	1854 (59.6%)
Missing count	290	244
Disease relapse index		
Low	881 (26.3%)	178 (5.3%)
Int	2096 (62.7%)	1247 (37.2%)
High	227 (6.8%)	1562 (46.5%)
Very high	141 (4.2%)	369 (11.0%)
Hematological malignancies		
Acute myeloid leukemia		2011 (59.9%)
Acute lymphoblastic leukemia		502 (15.0%)
Myelodysplastic syndrome (MDS)		264 (7.9%)
Myeloproliferative neoplasm (MPN)		179 (5.3%)
Chronic myeloid leukemia		82 (2.4%)
MDS & MPN		81 (2.4%)
Non-Hodgkins lymphoma		66 (2.0%)
Hodgkins lymphoma		59 (1.8%)
Multiple myeloma		57 (1.7%)
Chronic lymphatic leukemia		55 (1.6%)
Myeloablative conditioning		
No	1207 (36.4%)	1828 (56.1%)
Yes	2110 (63.6%)	1432 (43.9%)
Missing count	39	96
Total body irradiation		
No	2446 (73.0%)	2543 (76.6%)
Yes	905 (27.0%)	778 (23.4%)
Missing count	5	35
GVHD prevention regimen		
Cyclosporine A + MMF based	1457 (45.0%)	1223 (38.7%)
Cyclosporine A + MTX based	936 (28.9%)	705 (22.3%)
Cyclosporine A based	393 (12.1%)	383 (12.1%)
Tacrolimus/Sirolimus + MMF based	242 (7.5%)	538 (17.0%)
Tacrolimus/Sirolimus + MTX based	48 (1.5%)	44 (1.4%)
Tacrolimus/Sirolimus based	58 (1.8%)	99 (3.1%)
Other	103 (3.2%)	168 (5.3%)
Missing count	119	196
In vivo T cell depletion for second alloSCT		
No		1560 (48.6%)
Campath		142 (4.4%)
ATG		1506 (46.9%)
Donor to patient CMV positivity		
Positive to positive	893 (28.1%)	1395 (43.3%)
Positive to negative	653 (20.5%)	286 (8.9%)
Negative to positive	332 (10.4%)	766 (23.8%)
Negative to negative	1304 (41.0%)	772 (24.0%)
Missing count	174	137

*AlloSCT* allogeneic stem cell transplantation, *ATG* anti-T-cell globulin (also termed anti-thymocyte globulin), *CMV* cytomegaly virus, *MMF* mycophenolate mofetil, *MTX* methotrexate.

For first alloSCT, myeloablative conditioning was used in 64%. Most frequent donor types were identical siblings (39%), matched unrelated 10/10 (31%), and mismatched unrelated 9/10 (9%). A description of events and timelines between first- and second alloSCT is given in Table 2. Acute GVHD grades II-IV occurred in 16% and chronic GVHD occurred in 29% after first alloSCT. The median time from first alloSCT to second alloSCT was 1.73 years (Q1 0.93, Q3 3.41). The median time from relapse after first alloSCT to second alloSCT was 0.4 years (Q1 0.23, Q3 0.8).

**Table 2:** Description of events and timelines between first- and second alloSCT.

**Acute or Chronic GVH between first and second alloSCT**	
No	2128 (58.9%)
Yes	1207 (41.1%)
Missing count	422
**cGVH between first and second alloSCT**	
No	2130 (71.4%)
Yes	853 (28.6%)
Missing count	373
**cGVH Ext between first and second alloSCT**	
No	2621 (89.3%)
Yes	313 (10.7%)
Missing count	422
**aGVH-III/IV between first and second alloSCT**	
No	3133 (97.0%)
Yes	98 (3.0%)
Missing count	125
**aGVH-II/IV between first and second alloSCT**	
No	2701 (83.6%)
Yes	530 (16.4%)
Missing count	125
**Delay first to second alloSCT, years**	
Median [Q1, Q3]	1.7 (0.9, 3.4)
[Min, Max]	0.1–27.8
**Delay relapse to second alloSCT, years**	
Median [Q1, Q3]	0.4 (0.2, 0.8)
[Min, Max]	0.0–19.6

The median age at second alloSCT was 48 years. For second alloSCT, myeloablative conditioning was used in 44%. About 49% of patients who received myeloablative conditioning during their first alloSCT did not receive it at their second alloSCT. About 32% of those who did not receive myeloablative conditioning during their first alloSCT had received it during their second alloSCT. Most frequent donor types were matched unrelated 10/10 (35%), haploidentical (22%), and identical siblings (18%). Median follow-up was 3.7 [CI 95%: (3.4–4)] years after second alloSCT. GVHD prophylaxis regimens are shown in Table 1. 79.1% of patients receiving second alloSCT from haploidentical donors received post-transplantation Cyclophosphamide as GVHD prophylaxis.

### NRM, survival and relapse incidence after second alloSCT

**Fig. 1:**
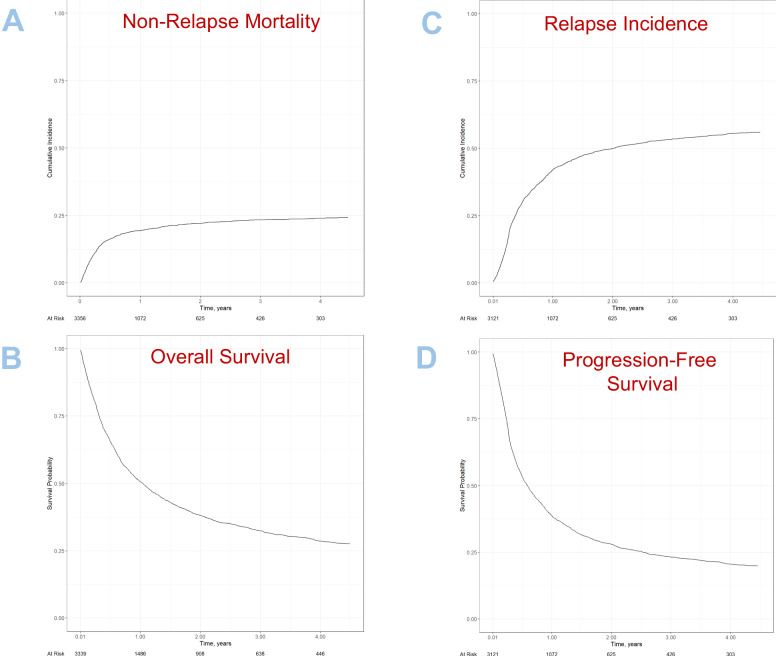
Survival outcomes and relapse after second alloSCT. **A** Non-relapse mortaility, **B** Overalll survival, **C** Relapse incidence, **D** Progression-free survival.

Univariate outcomes are shown in Fig. 1 and Table 3. The results of the multivariate analyses are summarized in Table 4.

**Table 3:** Univariate outcomes.

	Incidence % (CI 95%)
Non-relapse mortality	22.1 (20.6–23.6)
Relapse incidence	50 (48.1–51.8)
Overall survival	38.2 (36.4–39.9)
Progression-free survival	28 (26.3–29.7)
Acute GVHD-II/IV	23.8 (22.2–25.4)
Acute GVHD-III/IV	10.3 (9.3–11.5)
Chronic GVHD	29.9 (28.2–31.6)
Extensive chronic GVHD	13.6 (12.4–15)

All outcomes except acute GVHD are given at two years after second alloSCT. Acute GVHD is given at day +100 after second alloSCT.

**Table 4:** Multivariate analysis of survival and relapse related outcomes after second alloSCT.

Characteristic	Non relapse mortality (NRM)		Relapse incidence (RI)		Overall survival (OS)		Progression-free survival (PFS)	
	HR (95% CI)^a^	*p*-value^b^	HR (95% CI)^a^	*p*-value^b^	HR (95% CI)^a^	*p*-value^b^	HR (95% CI)^a^	*p*-value^b^
**Patient sex at birth** (Female vs. Male)	0.83 (0.69–1.00)	0.048*	0.96 (0.84–1.09)	0.51	0.90 (0.81–1.01)	0.066	0.91 (0.82–1.01)	0.088
**Donor sex at birth** (Female vs. Male)	1.14 (0.94–1.38)	0.17	0.96 (0.84–1.09)	0.53	1.07 (0.95–1.20)	0.26	1.01 (0.91–1.13)	0.84
**Age at HSCT2**, 5 yrs inc	1.07 (1.04–1.11)	<0.001***	1.02 (1.00–1.05)	0.044*	1.06 (1.04–1.08)	<0.001***	1.04 (1.02–1.06)	<0.001***
**Donor type** (Ref: MSD)								
Haplo	1.50 (1.02–2.21)	0.039*	0.66 (0.51–0.84)	<0.001***	1.03 (0.83–1.27)	0.81	0.84 (0.68–1.03)	0.10
Unrelated	1.43 (1.01–2.02)	0.043*	0.75 (0.61–0.92)	0.006**	1.05 (0.87–1.26)	0.64	0.90 (0.76–1.07)	0.24
**Same donor in HSCT1-HSCT2** (Yes vs. No)	0.94 (0.71–1.24)	0.65	1.04 (0.86–1.26)	0.67	0.94 (0.79–1.10)	0.42	1.02 (0.87–1.20)	0.79
**Cell source** (BM vs. PB)	0.92 (0.64–1.33)	0.66	1.07 (0.83–1.38)	0.61	0.99 (0.80–1.22)	0.90	1.02 (0.82–1.26)	0.88
**GVHD within HSCT1-Relapse1** (Yes vs. No)	1.28 (1.07–1.54)	0.009**	1.06 (0.93–1.20)	0.42	1.20 (1.07–1.34)	0.001**	1.12 (1.01–1.25)	0.038*
**Delay HSCT1-Relapse1**, yrs	0.94 (0.90–0.98)	0.002**	0.90 (0.87–0.93)	<0.001***	0.91 (0.89–0.94)	<0.001***	0.91 (0.89–0.94)	<0.001***
**Delay Relapse1-HSCT2**, yrs	1.01 (0.96–1.07)	0.71	0.95 (0.91–1.00)	0.040*	0.95 (0.91–0.99)	0.008**	0.97 (0.94–1.01)	0.15
**Karnofsky** (≥90 vs. <90)	0.64 (0.53–0.77)	<0.001***	0.89 (0.78–1.02)	0.085	0.73 (0.65–0.81)	<0.001***	0.80 (0.72–0.89)	<0.001***
**Disease risk index (DRI)** [Ref: Low]	—		—		—		—	
Int	1.37 (0.88–2.13)	0.17	1.50 (1.06–2.13)	0.023*	1.40 (1.05–1.88)	0.023*	1.45 (1.10–1.92)	0.008**
High	1.48 (0.95–2.30)	0.083	2.31 (1.64–3.26)	<0.001***	1.92 (1.44–2.56)	<0.001***	2.00 (1.52–2.62)	<0.001***
Very high	1.97 (1.20–3.23)	0.007**	3.33 (2.29–4.84)	<0.001***	2.92 (2.13–3.98)	<0.001***	2.82 (2.09–3.81)	<0.001***
**Transplant year**, 5 yrs inc	0.82 (0.70–0.96)	0.013*	0.99 (0.88–1.10)	0.80	0.89 (0.81–0.98)	0.019*	0.93 (0.85–1.02)	0.11
**Donor to patient CMV positivity** [Ref: Pos to Pos]	—		—		—		—	
Pos to Neg	0.78 (0.55–1.12)	0.18	1.10 (0.87–1.38)	0.42	1.06 (0.87–1.28)	0.59	0.99 (0.81–1.19)	0.89
Neg to Pos	1.07 (0.85–1.33)	0.57	1.07 (0.91–1.26)	0.39	0.99 (0.86–1.13)	0.88	1.07 (0.94–1.22)	0.31
Neg to Neg	0.79 (0.62–1.01)	0.056	0.99 (0.84–1.16)	0.87	0.88 (0.76–1.01)	0.064	0.92 (0.80–1.05)	0.22
**Total body irradiation** (Yes vs. No)	0.67 (0.53–0.84)	<0.001***	0.99 (0.85–1.15)	0.91	0.93 (0.82–1.05)	0.24	0.88 (0.77–1.00)	0.042*
**Myeloablative conditioning** (Yes vs. No)	1.04 (0.87–1.25)	0.67	0.93 (0.81–1.06)	0.26	1.04 (0.93–1.17)	0.48	0.96 (0.86–1.07)	0.44

^a^*HR* hazard ratio, *CI* confidence interval.

^b^*p < 0.05; **p < 0.01; ***p < 0.001.

All known potential risk factors were included in the multivariate models: patient age, year of transplant, patient and donor gender, donor to patient CMV combination, Disease Risk Index (DRI), Karnofsky Performance Status (KPS), Donor Type, Same Donor in 1st alloSCT, Stem Cell Source, GVHD between 1st and 2nd alloSCT, Delay 1st alloSCT to 1st Relapse, Delay 1st Relapse to 2nd alloSCT, any level of total body irradiation (TBI), conditioning intensity (RIC vs. MAC). Center effect was taken into account by introducing a random effect or ‘frailty’ into all models. All factors were assessed at 2nd HSCT.

Statistically significant values are highlighted in bold numbers.

In multivariate analysis, several risk factors for NRM (2 years incidence: 22% [95% CI 20.6–23.6]) were identified: (a) the time of the second HSCT, with a HR of 1.07 [CI 95%: (1.04–1.11); *p* < 0.001] for every five-year increase; (b) low Karnofsky performance score [≥90 vs <90; HR: 0.64, CI 95%: (0.53–0.77); *p* < 0.001]; (c) sex—with females exhibiting a lower risk than males [HR: 0.83, CI 95%: (0.69–1); *p* = 0.048]; (d) donor type—with haploidentical donors [HR: 1.50, CI 95%: (1.02–2.21); *p* = 0.039] and unrelated donors (UD) [HR: 1.43, CI 95%: (1.01–2.02); *p* = 0.043] showing higher risks compared to matched sibling donors; (e) previous GVHD before the second alloSCT increased NRM risk [HR: 1.28, CI 95%: (1.07 to 1.54); *p* = 0.009]; (f) a very high DRI significantly elevated the NRM risk [HR: 1.97, CI 95%: (1.20–3.23); *p* = 0.007] relatively to Low DRI.; (g) longer delays from first alloSCT to relapse were associated with a decreased NRM risk [HR:0.94, CI 95%: (0.90 to 0.98); *p* = 0.002] for every year increase, as was recent years of transplant [HR {5 years increment}: 0.82, CI 95%: (0.70–0.96); *p* = 0.013]; and (h) TBI was also associated with a decreased risk of NRM [HR: HR:0.67, CI 95%: (0.53 to 0.84); *p* < 0.001].

RI was 50% [95% CI 48.1–51.8] at 2 years after second alloSCT and a high DRI relative to low was an important risk factor for relapse [HR {very high vs. low}: 3.33, CI 95%: (2.29–4.84); *p*-value < 0.001] and [HR {high vs. low}: 2.31, CI 95%: (1.64–3.26); *p* < 0.001]. OS was 38% [95% CI 36.4–39.9] and PFS 28% [95% CI 26.3–29.7] at 2 years after second alloSCT (Fig. 1, Table 3). Risk factors for reduced OS and PFS after second alloSCT were roughly similar to those described for NRM (Table 4).

Relapse of the underlying malignancy was the most frequent cause of death after second alloSCT, accounting for 63% (*n* = 1347) of total deaths. The median time to relapse after second alloSCT was 145 days (Q1, Q3 80, 325) [Min, Max 2–2408]. NRM causes of death were: infections 16% (*n* = 334), GVHD 11% (*n* = 232), and other alloSCT-related causes 7% (*n* = 144) of total deaths. Secondary malignancies contributed to ~0.5% of total deaths.

### GVHD after second alloSCT

**Fig. 2:**
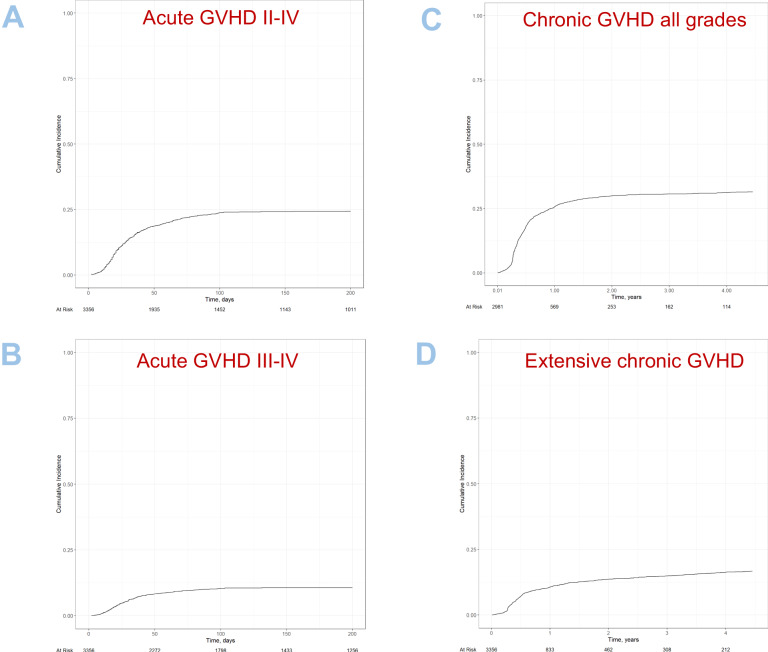
GVHD outcomes after second alloSCT. **A** aGVHD Grades II-IV, **B** aGVHD Grades II-IV, **C** cGVHD all grades, **D** extensive cGVHD.

Univariate outcomes are shown in Fig. 2 and Table 3. The results of the multivariate analyses are summarized in Table 5.

**Table 5:** Multivariate analysis of GVHD related outcomes after second alloSCT.

Characteristic	Chronic GVHD all grades		Extensive chronic GVHD		Acute GVHD II-IV		Acute GVHD III-IV	
	HR (95% CI)^a^	*p*-value^b^	HR (95% CI)^a^	*p*-value^b^	HR (95% CI)^a^	*p*-value^b^	HR (95% CI)^a^	*p*-value^b^
**Patient sex at birth** (Female vs. Male)	0.98 (0.83–1.16)	0.79	0.92 (0.73–1.16)	0.49	0.93 (0.77–1.11)	0.41	0.76 (0.58–1.00)	0.054
**Donor sex at birth** (Female vs. Male)	1.21 (1.01–1.43)	0.034*	1.09 (0.85–1.39)	0.50	1.22 (1.01–1.47)	0.036*	1.29 (0.98–1.70)	0.072
**Age at HSCT2**, 5 yrs inc	0.99 (0.96–1.02)	0.58	0.98 (0.94–1.03)	0.46	0.96 (0.93–1.00)	0.024*	0.96 (0.91–1.00)	0.078
**Donor type** (Ref: MSD)	—		—		—		—	
Haplo	0.60 (0.44–0.84)	0.003**	0.61 (0.38–0.97)	0.036*	1.28 (0.90–1.84)	0.17	1.26 (0.75–2.10)	0.38
Unrelated	0.67 (0.51–0.88)	0.003**	0.72 (0.49–1.04)	0.078	1.27 (0.93–1.73)	0.13	1.08 (0.69–1.69)	0.73
**Same donor in HSCT1-HSCT2** (Yes vs. No)	0.91 (0.70–1.18)	0.47	0.92 (0.65–1.31)	0.66	1.10 (0.84–1.43)	0.48	1.23 (0.84–1.80)	0.29
**Cell source** (BM vs. PB)	0.95 (0.66–1.36)	0.78	0.81 (0.47–1.40)	0.45	0.77 (0.53–1.13)	0.18	0.59 (0.31–1.12)	0.11
**GVHD within HSCT1-Relapse1** (Yes vs. No)	1.60 (1.35–1.89)	<0.001***	1.83 (1.45–2.31)	<0.001***	1.51 (1.26–1.81)	<0.001***	1.36 (1.03–1.79)	0.029*
**Delay HSCT1-Relapse1**, yrs	0.98 (0.95–1.01)	0.27	0.96 (0.92–1.00)	0.077	0.94 (0.90–0.98)	0.003**	0.89 (0.82–0.96)	0.002**
**Delay Relapse1-HSCT2**, yrs	0.97 (0.92–1.02)	0.21	0.95 (0.88–1.03)	0.22	0.99 (0.93–1.05)	0.68	0.98 (0.90–1.08)	0.73
**Karnofsky** (≥90 vs. <90)	0.93 (0.77–1.11)	0.40	0.81 (0.63–1.03)	0.086	0.73 (0.61–0.88)	0.001**	0.70 (0.53–0.93)	0.014*
**Disease risk index (DRI)** [Ref: Low]	—		—		—		—	
Int	0.98 (0.69–1.40)	0.92	1.37 (0.81–2.31)	0.24	0.60 (0.42–0.87)	0.006**	0.67 (0.38–1.18)	0.17
High	1.08 (0.76–1.53)	0.67	1.41 (0.84–2.36)	0.20	0.71 (0.50–1.02)	0.061	0.81 (0.46–1.41)	0.45
Very high	1.23 (0.81–1.88)	0.34	1.58 (0.85–2.92)	0.15	0.71 (0.47–1.09)	0.12	0.96 (0.51–1.81)	0.91
**Transplant year**, 5 yrs inc	0.87 (0.75–1.00)	0.055	1.10 (0.89–1.36)	0.36	1.03 (0.88–1.20)	0.75	1.07 (0.84–1.36)	0.58
**Donor to patient CMV positivity** [Ref: Pos to Pos]	—		—		—		—	
Pos to Neg	1.14 (0.85–1.54)	0.38	1.16 (0.79–1.71)	0.45	1.03 (0.73–1.44)	0.87	1.02 (0.61–1.72)	0.93
Neg to Pos	0.92 (0.74–1.15)	0.47	0.85 (0.62–1.15)	0.29	1.11 (0.89–1.39)	0.37	1.32 (0.95–1.82)	0.10
Neg to Neg	1.10 (0.89–1.35)	0.38	0.85 (0.63–1.14)	0.27	1.08 (0.86–1.35)	0.52	0.92 (0.64–1.32)	0.63
**Total body irradiation** (Yes vs. No)	1.10 (0.91–1.33)	0.32	1.00 (0.76–1.30)	0.98	1.20 (0.98–1.46)	0.079	1.08 (0.80–1.47)	0.61
**Myeloablative conditioning** (Yes vs. No)	1.0 (0.83–1.19)	0.95	1.13 (0.88–1.45)	0.34	0.97 (0.80–1.17)	0.76	1.04 (0.79–1.38)	0.78

^a^*HR* hazard ratio, *CI* confidence interval.

^b^*p < 0.05; **p < 0.01; ***p < 0.001.

All known potential risk factors were included in the multivariate models: patient age, year of transplant, patient and donor gender, donor to patient CMV combination, Disease Risk Index (DRI), Karnofsky Performance Status (KPS), Donor Type, Same Donor in 1st alloSCT, Stem Cell Source, GVHD between 1st and 2nd alloSCT, Delay 1st alloSCT to 1st Relapse, Delay 1st Relapse to 2nd alloSCT, any level of total body irradiation (TBI), conditioning intensity (RIC vs. MAC). Center effect was taken into account by introducing a random effect or ‘frailty’ into all models. All factors were assessed at 2nd HSCT.

Statistically significant values are highlighted in bold numbers.

The cumulative incidences of acute GVHD grades II–IV and III–IV at 100 days post second alloSCT were 24% [95% CI 22.2–25.4] and 10% [95% CI 9.3–11.5], respectively (Fig. 2, Table 3). In multivariate analysis, we identified several risk factors for acute GVHD: (a) the occurrence of any type of GVHD after first alloSCT, for grades II/IV [HR: 1.51, CI 95%: (1.26–1.81); *p* < 0.001], for grades III/IV [HR: 1.36, CI 95%: (1.03–1.79); *p* = 0.029]; (b) shorter delays from first alloSCT to first relapse, for grades II/IV [[HR: 0.94, CI 95%: (0.90–0.98); *p* = 0.003]] for every year of increase, for grades III/IV [HR: 0.89, CI 95%: (0.82–0.96); *p* = 0.002] for every year of increase; (c) low Karnofsky performance score, for grades II/IV [HR {≥90 vs. <90}: 0.73, CI 95%: (0.61–0.88); *p* = 0.001], for grades III/IV [HR {≥90 vs. <90}: 0.70, CI 95%: (0.53–0.93); *p* = 0.014] (Table 5).

The cumulative incidences of overall chronic GVHD and of extensive chronic GVHD at 2 years post alloSCT were 30% [95% CI 28.2–31.6] and 14% [95% CI 12.4–15], respectively (Fig. 2, Table 3). In multivariate analysis, we identified some risk factors for the development of chronic GVHD: The occurrence of any type of GVHD before second alloSCT and the type of donor used. Specifically, experiencing GVHD before the second alloSCT significantly increases the risk for chronic GVHD [HR: 1.60, CI 95%: (1.35–1.89); *p* < 0.001] and for extensive chronic GVHD [HR: 1.83, CI 95%: (1.45–2.31); *p* < 0.001].

### Donor choice for second alloSCT

Regarding the donor choice for second alloSCT, both haploidentical and UD in second alloSCT were beneficial as compared to identical sibling donors. However, NRM was lower in recipients of unrelated donor grafts that were human leukocyte antigen (HLA)-mismatched vs. HLA-matched (Supplementary Tables 1 and 2). The use of haploidentical donors was associated to a lower risk of all grades chronic GVHD [HR: 0.60, CI 95%: (0.44–0.84); *p* = 0.003] compared to the use of identical-sibling donors. This effect was consistent for extensive chronic GVHD [0.61, CI 95%: (0.38–0.97); *p* = 0.036]. Furthermore, use of UD was associated with a reduced risk of all grades chronic GVHD [HR: 0.67, CI 95%: (0.51–0.88); *p* = 0.003] compared to the use of matched-sibling donors (Table 5). The lower GVHD rates in unrelated and haploidentical donors are possibly explained by different GVHD prophylaxis regimens: in our study 66% of patients receiving unrelated second alloSCT vs. 21% of patients receiving identical sibling second alloSCT received in vivo T cell depletion with ATG (Anti-T cell/anti-thymocyte globulin). In addition, most haploidentical alloSCTs were performed with post-transplantation Cyclophosphamide. Of note, the donor age was closely related to the donor type: in identical sibling donors the median age was 47.3 years (Q1, Q3 34.6, 56) vs. 38 years (Q1, Q3 27.7, 51.1) in haploidentical donors vs, 30.2 years (Q1, Q3 24.9, 37.6) in UD.

In 20% of cases the same stem cell donor was used in first- and second alloSCT and in 80% of cases a different donor was chosen. In multivariate analysis, the use of the same alloSCT donor for second alloSCT vs. a different donor was not associated with any of the survival endpoints or relapse (Table 4). Furthermore, use of the same vs. a different second alloSCT donor was not associated with the incidence or severity of GVHD (Table 5).

## Discussion

This recent EBMT analysis of second alloSCT reports more patients as previous publications on the same topic [2–4, 9–12]. We found that NRM after second alloSCT was considerably lower as compared to previous cohorts [1–4, 9–12] but higher than NRM after first alloSCT [13–17].

The most important limitation of our registry data is the lack of information about how centers selected patients for second alloSCT. The current standard of care is to base second alloSCT decisions on the most important factors, including: (a) extent and aggressiveness of relapsed disease, including the presence of extramedullary involvement and cytogenetic/molecular risk factors; (b) patient’s overall health, organ function, and ability to tolerate conditioning regimens; (c) the duration between the first transplant and relapse; (d) availability of suitable donors; and (e) pre-existing comorbidities as well as prior transplant-related toxicities. The current dataset on second alloSCTs is probably a positive selection based on the above factors.

The 2 years PFS of 28% that we have observed in our study can be interpreted from different viewpoints. The positive view is that this is a good outcome for an extremely high-risk population for mortality and for relapse. The more negative view is the relatively low survival in this selected population of patients who received a second alloSCT. To better answer the question if a second alloSCT is preferable to an alternative therapy in a given situation, results from randomized prospective clinical studies are needed. These studies should include more extensive data on morbidity as well as patient-reported outcomes. OS, while a clear endpoint may underrepresent the morbidity associated with second alloSCT. In addition, we found that survival continues to fall after 2 years and thus longer-term follow-up may be needed to ultimately determine the successes of second alloSCTs. However, currently no such studies are underway and clinical decision-making has to be based on the available evidence.

### Comparison of NRM to previous reports

In a previous report on second alloSCTs during the period of 1994–2009 the cumulative incidence of NRM was 33% at 1 year and 40% at 5 years. The lower NRM we found in our recent cohort is in line with a previous report of first alloSCT also finding a considerable reduction of NRM over time [16]. Our data needs to be put in perspective with reports on second alloSCTs in previous disease-specific analyses [2–4, 9–12, 18]. One example is a report on adult patients with acute leukemia who received a second reduced-intensity alloSCT between 2000 and 2012 [4]. At 2 years, the cumulative incidence of NRM was with 22% similar to our current data. However, OS at 2 years was low with 22%, with relapse being the main factor. This is in line with our study showing 50% relapse at 2 years after 2nd alloSCT. A recent report included acute leukemia patients receiving a second haploidentical alloSCT and found a 2 years NRM of 18% and OS of 34% [9]. This is in line with another recent publication showing 19% NRM at two years in *n* = 80 leukemia patients after second alloSCT [19].

NRM after second alloSCT compares unfavorably to NRM rates after first alloSCT as data from prospective studies [13–15] and registry analyses demonstrates [16]. A recent prospective study found 1 year NRM of 11% after first alloSCT in acute leukemia patients [17]. A registry analysis found 12% NRM in patients with hematologic malignancies undergoing the first alloSCT from a matched-related donor [16]. When looking at the reasons of death for NRM in the published studies of first alloSCT and in our current study on second alloSCT it becomes evident that there are no major differences with infections, GVHD, and other alloSCT-related causes being the main factors.

### GVHD outcomes comparison and risk factor

A recent study in patients with hematologic malignancies undergoing a first alloSCT between 2011 and 2015 found a cumulative incidence of acute GVHD grades II–IV and III–IV of 28% and 11%, respectively [20]. These data is in a similar range of what we found after second alloSCT and suggest that the acute GVHD risk is not increased after second alloSCT. However, we found that the acute GVHD risk after second alloSCT mainly depends on the fact if GVHD (acute or chronic) occurred after first alloSCT. The presence of GVHD after first alloSCT as main risk factor for acute GVHD after second alloSCT has been described previously [1].

### Influence of the same donor vs. different donor in second alloSCT

When the decision is made to perform a second alloSCT in patients with relapse of the hematologic malignancy after first alloSCT there is one difficult other decision to make: which donor to choose? Most physicians tend to choose a different donor from the one used in first alloSCT. Our current results confirm this clinical preference. One major reason why a different donor is used as opposed to the same donor that has already been used in first alloSCT is probably common sense, with the expectation that it may be better to change the donor when the use of the original donor has not resulted in cure of the underlying disease. However, we did not find any significant impact of second alloSCT donor selection on major outcomes. This aligns with the results of previous studies. Christopeit and co-workers found in *n* = 179 related or unrelated second alloSCT that selecting a new donor did not result in a relevant improvement nor deterioration of survival [21]. Ruutu et al. found that there was no difference in OS or relapse-free survival between transplantations from the same vs. another donor. The NRM was slightly lower and the relapse rate slightly higher (non-significant) when the donor was the same as at the first transplantation [1].

In conclusion, NRM and survival after second alloSCT in this recent EBMT cohort have shown considerable improvement when compared to historic reports. These data can be used as rationale for clinical decision-making on second alloSCT indications.

### Supplementary information

Supplement

## Data Availability

Individual participant data will not be shared because patients agreed to data sharing with EBMT as well as with publication of results, but not to share data with third parties.
