# Supplementary material for: How risky is a second allogeneic stem cell transplantation?
Source: Leukemia. 2024 Jun 25;38(8):1799–807. doi: 10.1038/s41375-024-02318-3 (PMC11286516; doi:10.1038/s41375-024-02318-3)
Supplement: Supplementary file 1 — Supplement [file 41375_2024_2318_MOESM1_ESM.docx]

**Suppl Table 1**. Multivariate analysis of survival and relapse related outcomes after second alloSCT excluding missing HLA UD donors. All known potential risk factors were included in the multivariate models: patient age, year of transplant, patient and donor gender, donor to patient CMV combination, Disease Risk Index (DRI), Karnofsky Performance Status (KPS),Donor Type, Same Donor in 1st alloSCT, Stem Cell Source, GVHD between 1st and 2nd alloSCT, Delay 1st alloSCT to 1st Relapse, Delay 1st Relapse to 2nd alloSCT, any level of total body irradiation (TBI), conditioning intensity (RIC vs. MAC). Center effect was taken into account by introducing a random effect or ‘frailty’ into all models. All factors were assessed at 2nd HSCT.

| Characteristic | **Non Relapse Mortality NRM** | | **Relapse Incidence RI** | | **Overall Survival OS** | | **Progression Free Survival PFS** | |
| --- | --- | --- | --- | --- | --- | --- | --- | --- |
|  | **HR** **(95% CI)***^1^* | **p-value***^2^* | **HR** **(95% CI)***^1^* | **p-value***^2^* | **HR** **(95% CI)***^1^* | **p-value***^2^* | **HR** **(95% CI)***^1^* | **p-value***^2^* |
| **Patient Sex at Birth**(Female vs. Male) | 0.82 (0.67 to 1.00) | 0.054 | 0.97 (0.85 to 1.11) | 0.68 | 0.92 (0.82 to 1.03) | 0.16 | 0.92 (0.82 to 1.03) | 0.15 |
| **Donor Sex at Birth**(Female vs. Male) | 1.12 (0.91 to 1.38) | 0.30 | 0.98 (0.85 to 1.13) | 0.76 | 1.08 (0.96 to 1.22) | 0.20 | 1.02 (0.91 to 1.14) | 0.76 |
| **Age at HSCT2**, 5 yrs inc | 1.08 (1.04 to 1.12) | **<0.001***** | 1.03 (1.00 to 1.05) | **0.043*** | 1.06 (1.04 to 1.09) | **<0.001***** | 1.04 (1.02 to 1.06) | **<0.001***** |
| **Donor Type** (Ref : MSD) |  |  |  |  |  |  |  |  |
| Haplo | 1.46 (0.98 to 2.18) | 0.062 | 0.66 (0.52 to 0.84) | **<0.001***** | 1.03 (0.82 to 1.28) | 0.81 | 0.84 (0.68 to 1.03) | 0.10 |
| UD Matched | 1.20 (0.83 to 1.72) | 0.34 | 0.75 (0.61 to 0.93) | **0.010**** | 0.98 (0.80 to 1.19) | 0.82 | 0.85 (0.71 to 1.03) | 0.092 |
| UD Mismatched | 1.68 (1.11 to 2.54) | **0.015*** | 0.81 (0.62 to 1.06) | 0.13 | 1.18 (0.94 to 1.49) | 0.15 | 1.01 (0.81 to 1.26) | 0.95 |
| **Same Donor in HSCT1-HSCT2**(Yes vs. No) | 0.95 (0.70 to 1.29) | 0.73 | 1.07 (0.88 to 1.29) | 0.51 | 0.96 (0.80 to 1.14) | 0.62 | 1.04 (0.89 to 1.23) | 0.60 |
| **Cell Source** (BM vs. PB) | 1.04 (0.70 to 1.54) | 0.85 | 1.16 (0.89 to 1.51) | 0.27 | 1.08 (0.86 to 1.35) | 0.52 | 1.12 (0.90 to 1.40) | 0.32 |
| **GVHD within HSCT1-Relapse1** (Yes vs. No) | 1.30 (1.06 to 1.59) | 0.013* | 1.07 (0.93 to 1.23) | 0.32 | 1.20 (1.06 to 1.35) | **0.004**** | 1.13 (1.01 to 1.27) | **0.034*** |
| **Delay HSCT1-Relapse1,** yrs | 0.92 (0.88 to 0.97) | **0.001**** | 0.89 (0.86 to 0.92) | **<0.001***** | 0.90 (0.87 to 0.93) | **<0.001***** | 0.90 (0.88 to 0.93) | **<0.001***** |
| **Delay Relapse1-HSCT2**, yrs | 1.02 (0.96 to 1.08) | 0.61 | 0.95 (0.90 to 1.00) | **0.036*** | 0.94 (0.90 to 0.98) | **0.004**** | 0.97 (0.93 to 1.01) | 0.14 |
| **Karnofsky**(>= 90 vs. < 90) | 0.62 (0.50 to 0.76) | **<0.001***** | 0.88 (0.77 to 1.02) | 0.081 | 0.72 (0.63 to 0.81) | **<0.001***** | 0.79 (0.70 to 0.89) | **<0.001***** |
| **Disease Risk Index (DRI)** [Ref : Low] |  |  |  |  |  |  |  |  |
| Int | 1.26 (0.75 to 2.12) | 0.38 | 1.46 (0.98 to 2.18) | 0.062 | 1.26 (0.91 to 1.75) | 0.17 | 1.40 (1.02 to 1.92) | **0.039*** |
| High | 1.45 (0.86 to 2.42) | 0.16 | 2.30 (1.55 to 3.41) | **<0.001***** | 1.78 (1.29 to 2.46) | **<0.001***** | 1.99 (1.45 to 2.72) | **<0.001***** |
| Very high | 1.74 (0.98 to 3.08) | 0.058 | 3.33 (2.19 to 5.07) | **<0.001***** | 2.65 (1.87 to 3.75) | **<0.001***** | 2.72 (1.94 to 3.82) | **<0.001***** |
| **Transplant Year**, 5 yrs inc | 0.81 (0.68 to 0.97) | **0.019*** | 0.97 (0.86 to 1.09) | 0.63 | 0.87 (0.79 to 0.97) | **0.013*** | 0.91 (0.83 to 1.01) | 0.077 |
| **Donor to Patient CMV positivity** [Ref : Pos to Pos] |  |  |  |  |  |  |  |  |
| Pos to Neg | 0.91 (0.62 to 1.33) | 0.61 | 1.10 (0.87 to 1.40) | 0.42 | 1.10 (0.89 to 1.35) | 0.39 | 1.04 (0.85 to 1.27) | 0.72 |
| Neg to Pos | 1.04 (0.82 to 1.33) | 0.73 | 1.02 (0.86 to 1.21) | 0.81 | 0.94 (0.81 to 1.09) | 0.44 | 1.03 (0.89 to 1.18) | 0.71 |
| Neg to Neg | 0.77 (0.59 to 1.01) | 0.059 | 0.98 (0.83 to 1.17) | 0.83 | 0.86 (0.73 to 1.00) | **0.045*** | 0.91 (0.79 to 1.06) | 0.22 |
| **Total Body Irradiation** (Yes vs. No) | 0.64 (0.50 to 0.82) | **<0.001***** | 1.02 (0.87 to 1.19) | 0.80 | 0.95 (0.82 to 1.09) | 0.43 | 0.89 (0.78 to 1.02) | 0.087 |
| **Myeloablative Conditioning**(Yes vs. No) | 1.04 (0.85 to 1.28) | 0.69 | 0.92 (0.80 to 1.06) | 0.25 | 1.06 (0.94 to 1.20) | 0.35 | 0.96 (0.85 to 1.08) | 0.49 |
| *^1^* HR = Hazard Ratio, CI = Confidence Interval; *^2^* *p<0.05; **p<0.01; ***p<0.001 | | | | | | | | |

**Suppl Table 2**. Multivariate analysis of GVHD related outcomes after second alloSCT excluding missing HLA UD donors. All known potential risk factors were included in the multivariate models: patient age, year of transplant, patient and donor gender, donor to patient CMV combination, Disease Risk Index (DRI), Karnofsky Performance Status (KPS),Donor Type, Same Donor in 1st alloSCT, Stem Cell Source, GVHD between 1st and 2nd alloSCT, Delay 1st alloSCT to 1st Relapse, Delay 1st Relapse to 2nd alloSCT, any level of total body irradiation (TBI), conditioning intensity (RIC vs. MAC). Center effect was taken into account by introducing a random effect or ‘frailty’ into all models. All factors were assessed at 2nd HSCT.

| Characteristic | **Chronic GVHD all grades** | | **Extensive chronic GVHD** | | **Acute GVHD II-IV** | | **Acute GVHD III-IV** | |
| --- | --- | --- | --- | --- | --- | --- | --- | --- |
|  | **p-value***^2^* | **HR** **(95% CI)***^1^* | **p-value***^2^* | **HR** **(95% CI)***^1^* | **p-value***^2^* | **HR** **(95% CI)***^1^* | **p-value***^2^* | **HR** **(95% CI)***^1^* |
| **Patient Sex at Birth**(Female vs. Male) | 1.01 (0.84 to 1.20) | 0.95 | 0.93 (0.73 to 1.18) | 0.55 | 0.97 (0.80 to 1.17) | 0.74 | 0.74 (0.55 to 0.98) | **0.038*** |
| **Donor Sex at Birth**(Female vs. Male) | 1.16 (0.97 to 1.39) | 0.11 | 1.09 (0.85 to 1.41) | 0.49 | 1.23 (1.01 to 1.49) | **0.038*** | 1.23 (0.92 to 1.65) | 0.16 |
| **Age at HSCT2**, 5 yrs inc | 1.00 (0.97 to 1.03) | 0.88 | 0.99 (0.95 to 1.03) | 0.59 | 0.96 (0.93 to 0.99) | **0.022*** | 0.95 (0.91 to 1.00) | 0.072 |
| **Donor Type** (Ref : MSD) |  |  |  |  |  |  |  |  |
| Haplo | 0.55 (0.39 to 0.77) | **<0.001***** | 0.56 (0.35 to 0.90) | **0.016*** | 1.21 (0.84 to 1.74) | 0.31 | 1.15 (0.69 to 1.92) | 0.59 |
| UD Matched | 0.62 (0.47 to 0.82) | **<0.001***** | 0.68 (0.46 to 1.00) | **0.048*** | 1.21 (0.88 to 1.67) | 0.24 | 0.78 (0.48 to 1.25) | 0.30 |
| UD Mismatched | 0.61 (0.42 to 0.87) | **0.007**** | 0.85 (0.52 to 1.39) | 0.53 | 1.36 (0.94 to 1.99) | 0.11 | 1.60 (0.96 to 2.67) | 0.070 |
| **Same Donor in HSCT1-HSCT2**(Yes vs. No) | 0.82 (0.63 to 1.08) | 0.16 | 0.86 (0.60 to 1.24) | 0.41 | 1.01 (0.76 to 1.33) | 0.95 | 1.13 (0.75 to 1.69) | 0.55 |
| **Cell Source** (BM vs. PB) | 0.96 (0.66 to 1.42) | 0.86 | 0.91 (0.52 to 1.61) | 0.76 | 0.80 (0.54 to 1.19) | 0.27 | 0.62 (0.33 to 1.19) | 0.15 |
| **GVHD within HSCT1-Relapse1** (Yes vs. No) | 1.56 (1.31 to 1.87) | **<0.001***** | 1.88 (1.47 to 2.41) | **<0.001***** | 1.46 (1.20 to 1.77) | **<0.001***** | 1.33 (0.99 to 1.78) | 0.058 |
| **Delay HSCT1-Relapse1,** yrs | 0.98 (0.95 to 1.01) | 0.28 | 0.96 (0.91 to 1.00) | 0.069 | 0.94 (0.89 to 0.98) | **0.004**** | 0.87 (0.80 to 0.95) | **0.001**** |
| **Delay Relapse1-HSCT2**, yrs | 0.97 (0.92 to 1.02) | 0.28 | 0.94 (0.87 to 1.03) | 0.19 | 0.99 (0.93 to 1.06) | 0.74 | 1.00 (0.91 to 1.10) | >0.99 |
| **Karnofsky**(>= 90 vs. < 90) | 0.92 (0.77 to 1.12) | 0.41 | 0.83 (0.65 to 1.08) | 0.16 | 0.70 (0.57 to 0.85) | **<0.001***** | 0.66 (0.49 to 0.88) | **0.005**** |
| **Disease Risk Index (DRI)** [Ref : Low] |  |  |  |  |  |  |  |  |
| Int | 0.88 (0.60 to 1.31) | 0.53 | 1.15 (0.66 to 2.01) | 0.62 | 0.55 (0.37 to 0.83) | **0.004**** | 0.54 (0.30 to 0.99) | **0.045*** |
| High | 1.01 (0.69 to 1.49) | 0.96 | 1.29 (0.74 to 2.24) | 0.37 | 0.66 (0.44 to 0.97) | **0.037*** | 0.61 (0.34 to 1.10) | 0.10 |
| Very high | 1.22 (0.77 to 1.92) | 0.40 | 1.41 (0.74 to 2.69) | 0.29 | 0.62 (0.39 to 1.00) | **0.048*** | 0.70 (0.36 to 1.36) | 0.29 |
| **Transplant Year**, 5 yrs inc | 0.89 (0.76 to 1.04) | 0.15 | 1.19 (0.95 to 1.48) | 0.13 | 1.03 (0.87 to 1.21) | 0.74 | 1.05 (0.82 to 1.35) | 0.69 |
| **Donor to Patient CMV positivity** [Ref : Pos to Pos] |  |  |  |  |  |  |  |  |
| Pos to Neg | 1.18 (0.86 to 1.61) | 0.31 | 1.10 (0.73 to 1.65) | 0.64 | 1.06 (0.74 to 1.51) | 0.76 | 1.16 (0.68 to 1.98) | 0.59 |
| Neg to Pos | 0.92 (0.73 to 1.16) | 0.47 | 0.77 (0.55 to 1.06) | 0.11 | 1.16 (0.91 to 1.47) | 0.22 | 1.40 (0.99 to 1.97) | 0.055 |
| Neg to Neg | 1.16 (0.93 to 1.44) | 0.18 | 0.80 (0.59 to 1.09) | 0.15 | 1.13 (0.88 to 1.43) | 0.34 | 1.01 (0.69 to 1.49) | 0.96 |
| **Total Body Irradiation** (Yes vs. No) | 1.12 (0.92 to 1.38) | 0.25 | 1.06 (0.80 to 1.40) | 0.69 | 1.28 (1.04 to 1.58) | **0.021*** | 1.11 (0.80 to 1.53) | 0.53 |
| **Myeloablative Conditioning**(Yes vs. No) | 1.01 (0.84 to 1.23) | 0.88 | 1.20 (0.92 to 1.55) | 0.18 | 0.98 (0.80 to 1.20) | 0.85 | 1.01 (0.75 to 1.35) | 0.97 |
| *^1^* HR = Hazard Ratio, CI = Confidence Interval; *^2^* *p<0.05; **p<0.01; ***p<0.001 | | | | | | | | |
